# Latent profiles analysis of Traditional Chinese Medicine Health Literacy in middle-aged and older adult patients with chronic diseases and its association with disease burden and self management

**DOI:** 10.3389/fpubh.2026.1775708

**Published:** 2026-05-05

**Authors:** Guoyin Han, Weiling Yang, Juan Wang, Jinfeng Deng, Ting Shi, Yinqin Zhong, Huihui Zhang

**Affiliations:** 1 Cardiovascular Department, Shenzhen Hospital (Futian) of Guangzhou University of Chinese Medicine, Shenzhen, China; 2 The Sixth Clinical Medical College of Guangzhou University of Traditional Chinese Medicine, Shenzhen, China; 3 President Office, Shenzhen Hospital (Futian) of Guangzhou University of Chinese Medicine, Shenzhen, China

**Keywords:** chronic disease, health literacy, latent profiles analysis, self-management, traditional Chinese medicine

## Abstract

**Objective:**

To examine the heterogeneity and determinants of Traditional Chinese Medicine Health Literacy (TCM-HL) among middle-aged and older adults patients with chronic diseases and its association with disease burden and self-management and provide evidence for targeted interventions.

**Methods:**

From August 2025 to November 2025, a total of 550 middle-aged and older adults patients with chronic diseases were recruited as convenience samples from a tertiary Chinese medicine hospital and its nine affiliated community health service centers located in Guangdong Province, China. Data were collected using structured questionnaires, which included the TCM-HL questionnaire, the Consumer Health Activation Index (CHAI), and the Brief Patient Experience with Treatment and Self-management (Brief PETS). Latent profile analysis (LPA) was conducted using M plus 8.3 software, while univariate and multivariate logistic regression analyses were performed using SPSS 27.0 software.

**Results:**

The average score of TCM-HL among middle-aged and older adults patients with chronic diseases was 62.80 ± 14.42, with an average item score of items was 1.70 ± 1.40. LPA identified three categories: C1 “Low TCM-HL” (*n* = 104, 20.68%); C2 “Moderate TCM-HL” (n = 220, 43.47%), and C3 ‘High-TCM-HL’ (*n* = 179, 35.59%). Multinomial logistic regression analysis indicated that marital status, occupation, per capita household income, medical insurance, the CHAI and the PETS are the significant influencing factors of TCM-HL in this populations (*p* < 0.05).

**Conclusion:**

The level of TCM-HL among middle-aged and older adults patients with chronic diseases is generally low, exhibiting significant heterogeneity across various subgroups. Therefore, targeted interventions that are tailored to the specific needs of each subgroup are crucial for enhancing TCM-HL within these populations.

## 1 Introduction

Chronic non-communicable diseases (NCDs), commonly referred to as chronic diseases, are complex conditions influenced by multiple factors, including physiological abnormalities, genetic predisposition, environmental influences and personal behaviors. Addressing these diseases comprehensively presents significant challenges, as they are the leading cause of morbidity and mortality worldwide (1). According to a 2018 report by the World Health Organization (WHO), approximately 41.1 million people globally died from chronic non-communicable diseases, accounting for 71% of all deaths. This figure is projected to rise to 52 million by 2030 (2). In China, over 180 million older adults individuals suffer from chronic non-communicable diseases, representing 75% of all chronic disease cases and 88.5% of total deaths (3, 4). The burden of disease in China is characterized by a high incidence of non-communicable diseases (5, 6), which is the most significant factor contributing to years lived with disability (7). Chronic diseases, including diabetes, cardiovascular diseases, chronic respiratory diseases and malignant tumors, pose major health threats to the population. These diseases are marked by insidious onset, complex etiologies, and prolonged duration, affecting multiple systems such as the endocrine, cardiovascular, and respiratory systems (8). This situation presents a serious public health challenge and adversely impacts national health and social and economic development. Non-communicable diseases necessitate continuous self-management and adherence to intricate treatment regimens. Disease self-management emerges as a promising strategy for managing chronic diseases, enabling patients to actively identify and address disease-related issues to prevent complications or mitigate disability (9). However, the self-management behaviors of older adults patients with chronic diseases are generally inadequate, which is associated with a lack of proactive health awareness and insufficient understanding of disease management (10). Health literacy is critically important for effective chronic disease management. Because studies indicate that inadequate health literacy correlates with poorer disease management skills (11), and more severe outcomes for patients with multiple chronic conditions (12–15). The underlying issue often stems from patients’ lack of disease awareness and psychological self-confidence, hindering their ability to effectively manage chronic disease risk factors (16). Health literacy interventions have proven effective in enhancing the health of patients with chronic diseases (17). This study found that (18) developing key health literacy skills is a more effective approach to enhancing self-management behavior, particularly for individuals with multiple chronic diseases. Lu (19) emphasized that adequate health literacy significantly improves health outcomes of older adults patients with chronic diseases. This underscores the critical role of health literacy in facilitating effective disease management and enhancing patient self-management. Consequently, fostering health literacy has emerged as a top priority for public health and policy research to manage non-communicable chronic diseases more effectively (20).

In the context of ‘Healthy China 2030’ (21), the significance of traditional medical systems in health management is highlighted. Traditional Chinese Medicine Health Literacy (TCM-HL) represents a distinctive form of health literacy that embodies traditional Chinese characteristics. It refers to the ability of individuals to acquire and comprehend information and services related to TCM health knowledge, and utilize this information and these services to make informed decisions that promote and maintain their health. TCM-HL reflects a person’s capability to understand and apply the health information based on TCM, including disease etiology, treatment modalities, and preventive practices (22). At present, there is a lack of literature exploring the impact of TCM-HL on disease management in patients with chronic diseases, which hampers our understanding of this vital area. A thorough investigation of the relationship between TCM-HL levels and the treatment burden experienced by patients with chronic diseases may contribute to improved disease management. Therefore, this study employed the Brief Patient Experience with Treatment and Self-management (Brief PETS) to evaluate the treatment experience and emphasize the burden of disease. The research hypothesis posits that higher Brief PETS scores—indicative of a heavier treatment burden and poorer self-management experience—may correlate with lower TCM-HL.

At present, most of the research on health literacy focuses on the level status (22, 23), assuming a homogeneous population and neglecting individual-level differences. This approach limits the identification of unique features and needs, hindering the development of personalized intervention strategies. Latent profile analysis (LPA) is a people-oriented method that can identify common response patterns, classify individuals into subgroups, and reveal potential group heterogeneity, thereby enhancing classification accuracy (24). To develop effective TCM-HL interventions, it is essential to acknowledge the heterogeneity within the target population. Consequently, this study analyzed the potential profile characteristics of TCM-HL among middle-aged and older adults individuals and above with chronic diseases, examining how different TCM-HL profiles affect patient treatment and self-management. Additionally, interventions were formulated to enhance TCM-HL in middle-aged and older adults patients with chronic conditions.

## 2 Methods

### 2.1 Study design

A cross-sectional study was conducted to assess TCM-HL in middle-aged and older adults patients with chronic diseases.

### 2.2 Participants

This study employed a convenience sampling method to recruit middle-aged and older adults patients with chronic diseases from August to November 2025. Participants were sourced from the inpatient department of a tertiary Chinese medicine hospital and its nine affiliated community health service centers located in Guangdong Province, China. Inclusion criteria were as follows: (1) age ≥45 years; (2) diagnosis of a chronic disease according to the ICD-10, including but not limited to hypertension, diabetes, cancer, chronic respiratory diseases, and coronary artery disease; (3) normal consciousness and the ability to read or communicate; and (4) voluntary participation with informed consent. Exclusion criteria included: (1) severe cognitive, speech, visual, auditory, or psychiatric impairments; (2) acute or critical illness with severe complications or organ failure during the study; (3) inability to engage in extended communication or having significant communication barriers; and (4) simultaneous participation in other related studies.

The calculation method of sample size in LPA mainly depends on three rules (25): (1)each subgroup has at least 50 samples; (2) the smallest subgroup accounted for at least 5% of the total sample; (3) The total sample size is at least 5 ~ 10 times the number of independent variables. In this study, we proposed 28 independent variables, which means that at least 280 samples are needed. Considering the loss rate of 20%, the adjusted sample size was 336 cases. The 503 participants we finally included satisfied this requirement and were consistent with the criteria that each group had at least 50 samples and a category probability of at least 5%. Ethical approval was obtained from the Ethics Committee of Shenzhen Hospital (Futian) of Guangzhou University of Chinese Medicine (Approval No. GZYLL(KY)-2025-009).

### 2.3 Research tools

#### 2.3.1 General information questionnaire

This questionnaire was developed by the research team through literature review and consultation with clinical experts. It collected data on demographics (e.g., age, gender, education level, marital status, occupation, per capita household income), and disease characteristics (e.g., type of chronic disease, self-assessed health).

#### 2.3.2 TCM-HL questionnaire

The Chinese Citizens’ Health Literacy Questionnaire of Traditional Chinese Medicine (2017 Edition) consists of five dimensions, including basic concepts of TCM, appropriate methods of public heath, TCM-based healthy lifestyle, health culture common sense of TCM and TCM information understanding ability (26). There were 37 questions in the questionnaire, with a total score of 100 points. The correct answer of the right/wrong and single-choice questions scored 2 points, and the multiple-choice questions scored 4 points. The higher the score, the higher the level of TCM-HL ≥ 80 points (80% of the total score) are considered to have sufficient TCM-HL. The scale was employed to assess the TCM-HL among Chinese residents. The Cronbach’s *α* coefficient of the questionnaire was 0.810, which verified satisfactory reliability and validity (27). The Cronbach’s *α* coefficient of the questionnaire in this study was 0.856, which was suitable for investigating the literacy level of middle-aged and older adults people.

#### 2.3.3 Consumer health activation index (CHAI)

The self-management ability of patients with chronic diseases is crucial for disease control and prognosis. Research has demonstrated that the level of positiveness effectively reflects the self-management capacity of these patients and serves as a predictor for their health outcomes (28, 29). Wolf developed the CHAI scale, which is publicly accessible, easy to manage, and score, making it suitable for individuals with varying levels of health literacy (30). The scale comprises three dimensions and ten items. A 6-point Likert scale was utilized, with scores ranging from 1 to 6, indicating “strongly disagree” to “strongly agree.” Scores from 0 to 79 are categorized as low positive, scores from 80 to 94 as moderate positive, and scores from 95 to 100 as high positive. The Cronbach’s *α* coefficient for the scale was found to be 0.812, while in our study, it was 0.890.

#### 2.3.4 The brief patient experience with treatment and self-management (brief PETS)

The scale is used to validate the patient’s treatment and self-management experience, especially for patients with chronic diseases (31, 32). The PETS scale is a Likert 5 scale, and the original scale has been validated in chronic disease populations in the United States and Europe (33). The Brief PETS was simplified on the basis of the source scale 2.0 version (31), with a total of 32 items and 11 dimensions. The scale can calculate the overall disease treatment burden score by summing the standardized scores across various dimensions. A higher score indicates a greater treatment burden either in a specific dimension or overall. This scale has been validated within the Chinese population (34, 35). The Cronbach’s *α* coefficient for the Chinese version of the Brief PETS scale is 0.914, while the Cronbach’s α coefficient reported in this study is 0.818.

### 2.4 Data collection

The data were from the inpatient department of a tertiary Chinese medicine hospital in Guangdong Province, China and its 9 affiliated community health centers. The sample included both inpatients and patients who were regularly followed up in the community or hospital for chronic disease management. Prior to recruitment, the research team received formal approval from the relevant departments of the hospital.

To enhance cultural and contextual sensitivity, investigators, who are trained clinical nurses, participate in routine care activities, enabling them to build rapport with patients and become familiar with their language habits and cultural backgrounds. Contact eligible participants in private and clearly explain the purpose, procedures, confidentiality measures and recording requirements of the study in demystifying language. Before registration, all participants were given written informed consent.

After obtaining consent, participants completed the questionnaire independently in a quiet environment. For those with visual, literacy, or physical difficulties, trained researchers provided one-on-one assistance to ensure comprehension without influencing responses. Additionally, researchers verified and supplemented disease-related information through a review of medical records. A total of 550 middle-aged and older adults patients were invited to participate, of whom 503 completed the questionnaire, resulting in a response rate of 91.45%. The dropout rate was 8.55%, primarily due to invalid responses, such as patterned answers (e.g., identical scores for all items), extreme values, or selecting multiple options in single-choice questions. Completed questionnaires were collected immediately after completion and checked for completeness on-site. Any missing or ambiguous answers were clarified with participants in real time. According to previous literature, a questionnaire was deemed invalid if it contained more than 10% patterned or extreme responses, or multiple answers to single-choice questions (36). To minimize expectation bias, participation was entirely voluntary, and respondents were informed that their answers would remain anonymous and would not affect their care. Researchers only assisted in providing the questionnaire or offering completion support (e.g., reading the questions) without influencing the content of the responses.

### 2.5 Statistical methods

Data were double-checked, organized, and entered into a database. Latent profile modeling was conducted in M plus 8.3 using the 5 TCM-HL dimensions as observed variables. Models with increasing class numbers were evaluated, and the optimal solution was selected based on both model fit and clinical comprehensibility. The evaluation criteria included: (1) Information criteria—AIC, BIC, and aBIC, where lower values indicate a better fit; (2) Classification accuracy—Entropy values (ranging from 0 to 1), with values closer to 1 indicating higher accuracy; and (3) Likelihood ratio tests-Lo–Mendell–Rubin (LMR) and bootstrap likelihood ratio test (BLRT), where a *p*-value<0.05 suggests that the K-class model outperforms the K − 1-class model. Common method bias was assessed using Harman’s single-factor test in SPSS 27.0. Skewness and kurtosis tested univariate normality; Mardia’s test assessed multivariate normality. Variables with a normal or approximately normal distribution are reported as Mean ± SD and compared using one-way ANOVA. Categorical variables are presented as frequencies or percentages and analyzed using the chi-square test or Fisher’s exact test. Variables that are significant in univariate analysis are included in multivariate logistic regression. A *p*-value of <0.05 is considered statistically significant.

## 3 Results

### 3.1 Common method bias test

Given the self-reported nature of the data, there exists a potential for common method bias. To mitigate this issue, participants were assured of their anonymity and confidentiality prior to the survey. During the survey, the order of items was randomized, and disease-related information was corroborated through multiple sources, including electronic medical records, to minimize single-source bias. Utilizing Harman’s single-factor test, we identified 15 factors with eigenvalues exceeding 1, with the first factor accounting for 14.554% of the variance. This value is lower than the 40% threshold (37), indicating that common method bias is not an important issue.

### 3.2 Sociodemographic characteristics of participants

**Figure 1:**
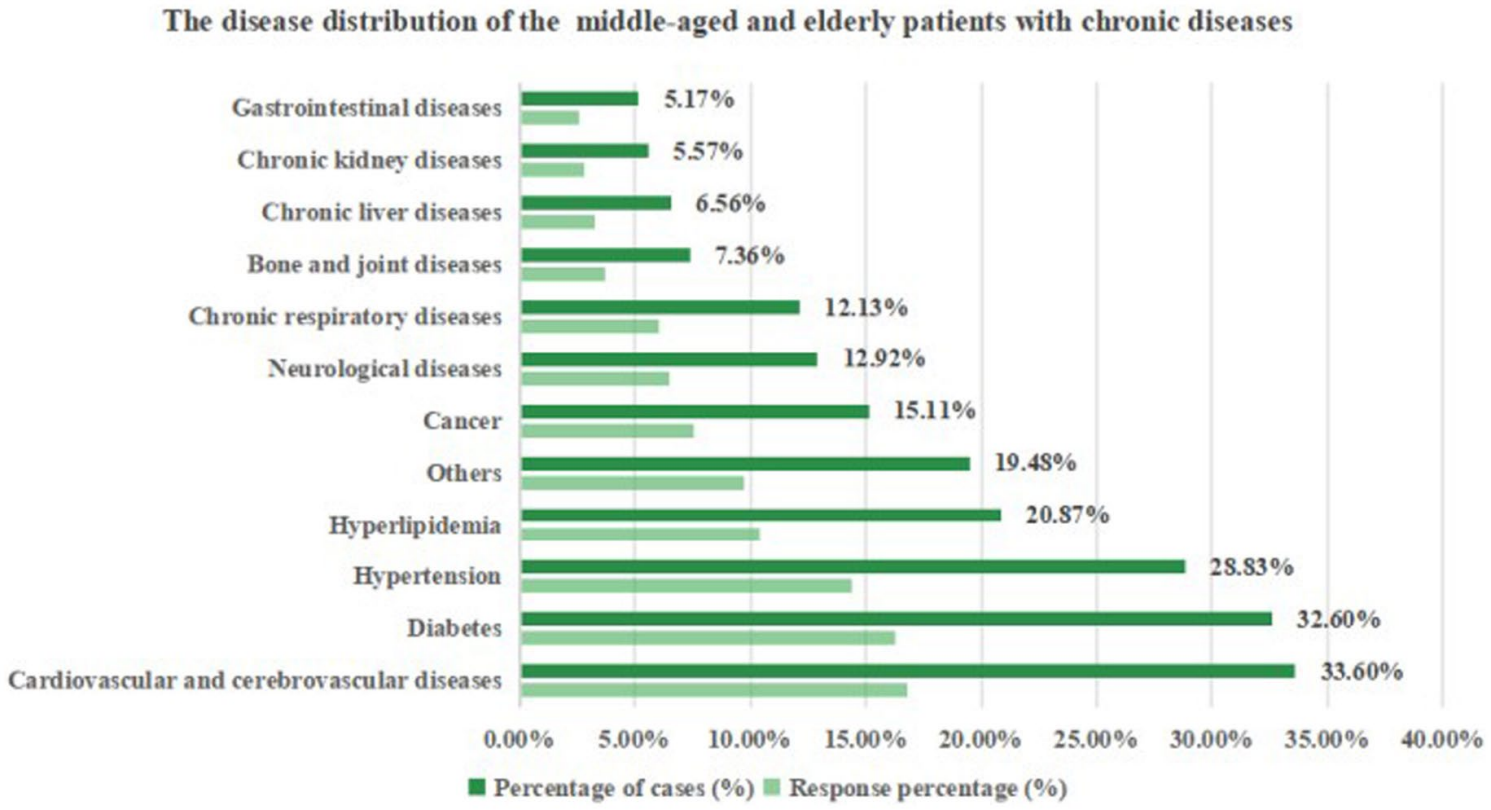
The disease distribution of the middle-aged and older adults patients with chronic diseases (*n* = 503).

A total of 503 participants took part in this study, with an average age of 54.75 ± 8.96 years. Among them, 67.59% were female. In terms of education, 63.82% had completed high school and above education; regarding marital status, 95.23% of participants were married; in terms of occupation, ‘others(including freelancers and flexible employment workers)’ (33.40%) and retirees (21.87%) accounted for the largest proportions. Most participants (89.26%) participated in a medical insurance plan; the majority had a monthly per capita household income of 5,000–9,999 (39.96%), and 46.52% of participants perceived their health status as average. As shown in Figure 1, cardiovascular and cerebrovascular diseases were the most common chronic diseases among participants (33.60%), followed by diabetes (32.60%). After describing and analyzing the number of illnesses of the respondents, it was found that there were 384 participants (76.34%) with 1–3 kinds of illnesses, and 71 participants (14.12%) with 4–6 kinds of chronic diseases. Specifically, the proportion of cardiovascular and cerebrovascular diseases, diabetes and hypertension was significantly higher, and the subjects generally had the problem of coexistence of multiple diseases.

### 3.3 Standard-reaching rate in TCM-HL

**Figure 2:**
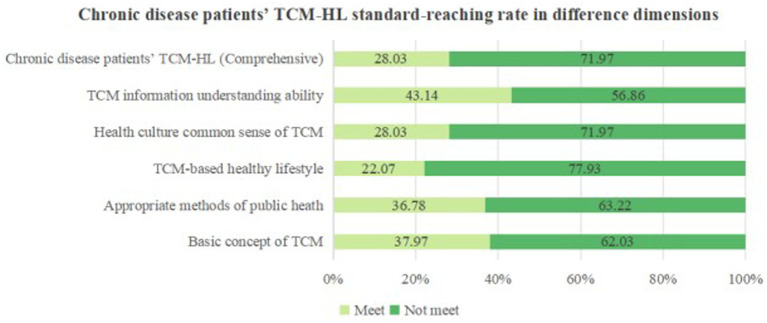
The compliance rate of TCM-HL in patients with chronic diseases.

Regarding TCM-HL, the overall score was 62.79 ± 14.42 points, with only 141 participants meeting the standard, representing 28.03% of the sample. Figure 2 illustrates that the highest compliance rate was observed in the TCM information understanding ability (43.14%), followed by the basic concept of TCM (37.97%) and the appropriate methods of public heath (36.78%). In contrast, the compliance rates for the health culture common sense of TCM (28.03%) and the TCM-based healthy lifestyle (22.07%) were notably low.

### 3.4 Latent profile analysis and profile naming

**Figure 3:**
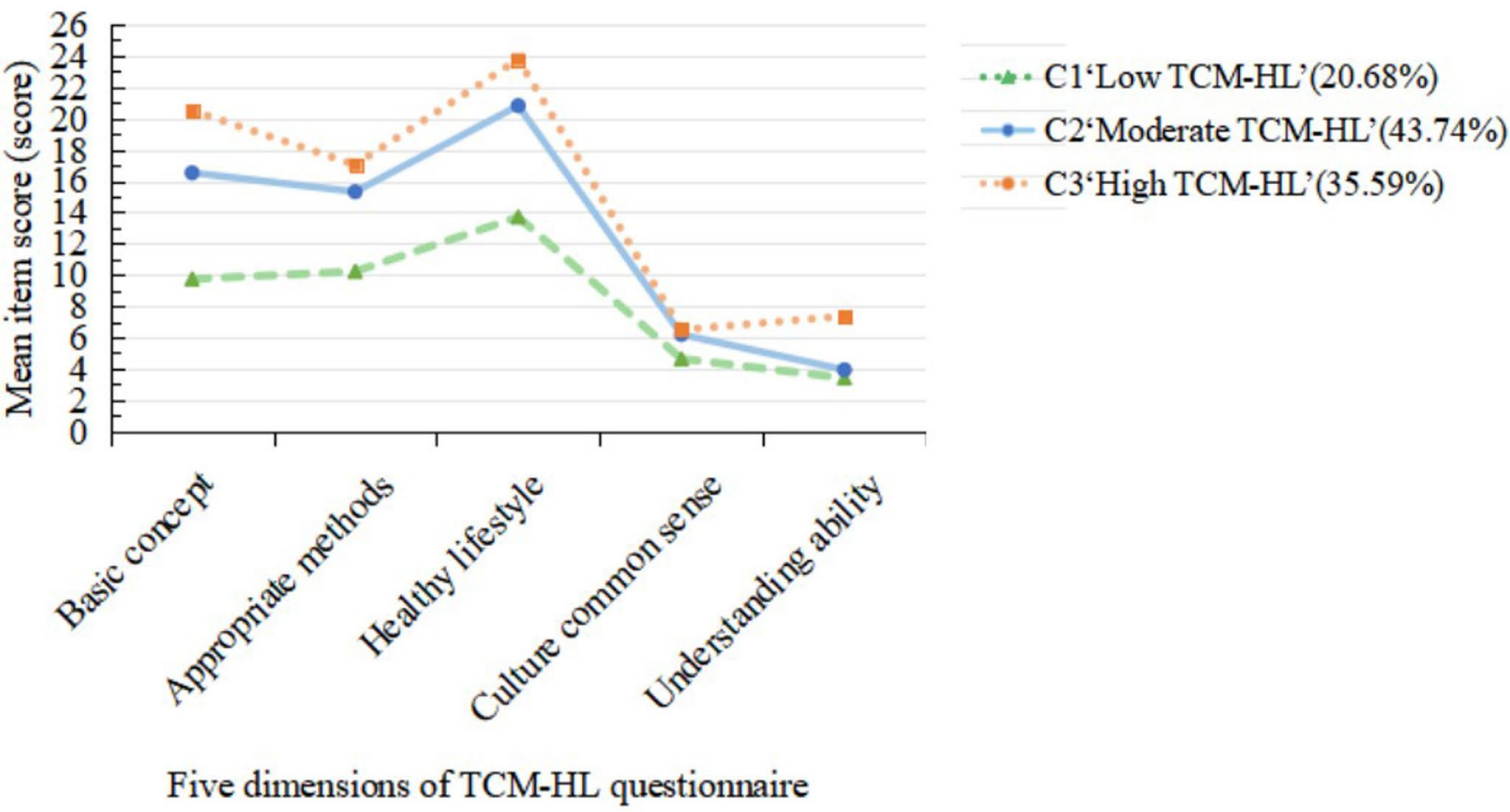
Latent profile chart of TCM-HL among middle-aged and older adults patients with chronic diseases.

The five dimensions of the TCM-HL scale were utilized as indicators, and LPA was conducted using M plus 8.3. Models with 1 to 5 categories (see Table 1) were evaluated. As the number of classes increased, the values of AIC, BIC and aBIC decreased, indicating an improvement in model fit. The entropy value exceeded 0.8, indicating high classification accuracy. In the three models, the entropy was recorded at 0.820, which was validated by LMR and BLART (*p* < 0.05). The number of participants in the category was 104, 220 and 179, respectively, with each exceeding 10% of the total sample. Considering the fitting indices, sample distribution and clinical relevance, three model types were selected. Table 2 presents the average scores of the three types of TCM-HL, while Figure 3 illustrates the vertical section, with these categories named based on the fluctuations of the average scores across the dimensions. The first category (C1) comprised 104 participants (20.68%), who exhibited the lowest scores across all dimensions, thus earning the designation “Low TCM-HL”. The second category (C2) included 220 participants (43.74%), whose scores across all dimensions were moderate, leading to the designation “Moderate TCM-HL”. The third category (C3) consisted of 179 participants (35.59%), who achieved higher scores in all dimensions, thereby being classified as ‘High TCM-HL’.

**Table 1:** The fitting results of the latent profile analysis models of TCM-HL in middle-aged and older adults patients with chronic diseases.

Trajectories	Log(L)	AIC	BIC	aBIC	Entropy	P		Class probability
						LMR	BLRT	
1	−7007.163	14034.325	14076.531	14044.790	—	—	—	1
2	−6831.188	13694.375	13761.905	13711.120	0.858	<0.001	<0.001	62.23%/37.78%
3	−6761.461	13566.923	13659.776	13589.946	0.820	<0.001	<0.001	20.68%/43.74%/35.59%
4	−6725.001	13506.003	13624.179	13535.305	0.825	<0.001	<0.001	40.95%/20.88%/28.23%/9.94%
5	−6697.797	13463.594	13607.094	13499.175	0.838	<0.001	0.0012	16.50%/5.17%/39.76%/9.94%/28.63%

**Table 2:** TCM-HL and scores of each dimension of the three potential profiles.

Variables	Number of items		Score	C1 (*n* = 104)	C2 (*n* = 220)	C3 (*n* = 179)
TCM-HL (Comprehensive)	37	a	62.80 ± 14.42	41.67 ± 7.26	62.74 ± 6.64	75.14 ± 9.39
		b	1.70 ± 1.40	1.13 ± 1.33	1.70 ± 1.38	2.03 ± 1.37
Basic concept of TCM	9	a	16.53 ± 4.99	9.73 ± 2.46	16.52 ± 2.99	20.49 ± 3.60
		b	1.84 ± 1.31	1.08 ± 1.27	1.84 ± 1.26	2.28 ± 1.18
Appropriate methods of public heath	8	a	14.87 ± 5.82	10.21 ± 6.27	15.31 ± 5.42	17.04 ± 4.34
		b	1.86 ± 1.63	1.28 ± 1.58	1.91 ± 1.60	2.13 ± 1.61
TCM-based healthy lifestyle	13	a	20.39 ± 5.93	13.69 ± 4.81	20.81 ± 3.65	23.77 ± 5.62
		b	1.57 ± 1.36	1.05 ± 1.27	1.60 ± 1.35	1.83 ± 1.34
Health culture common sense of TCM	4	a	5.97 ± 2.62	4.63 ± 2.42	6.18 ± 2.58	6.49 ± 2.53
		b	1.49 ± 1.20	1.16 ± 1.23	1.55 ± 1.18	1.62 ± 1.16
TCM infomation understanding ability	3	a	5.03 ± 2.07	3.40 ± 1.24	3.92 ± 1.23	7.35 ± 0.94
		b	1.68 ± 1.35	1.13 ± 1.11	1.31 ± 1.25	2.45 ± 1.25

a, total score; b, item mean score.

### 3.5 Univariate analysis of the latent profiles of TCM-HL in middle-aged and older adults patients with chronic diseases

Univariate analysis revealed significant differences in age, gender, education level, marital status, occupation, per capita household income, medical insurance, and self-assessed health (*p* < 0.05) (see Table 3). Additionally, significant differences (*p* < 0.05) were also observed in total and subscale scores for the Brief PETS and the CHAI across the three latent profiles (see Table 4).

**Table 3:** Univariate analysis of general characteristics and latent categories of TCM-HL among middle-aged and older adults patients with chronic diseases.

Variables		Number (%)	C1 (*n* = 104)	C2 (*n* = 220)	C3 (*n* = 179)	*χ*^2^/F	*p*-value
Age	45 ~ 55	321 (63.82)	47	150	124	42.745	<0.001
	55 ~ 65	123 (24.45)	28	60	35		
	>65	59 (11.73)	29	10	20		
Gender	Male	163 (32.41)	55	67	41	27.671	<0.001
	Female	340 (67.59)	49	153	138		
Education level	Primary school and below	34 (6.76)	24	10	0	124.503	<0.001
	Junior high school	100 (19.88)	36	161	131		
	High school and above	369 (73.36)	5	23	20		
Marital status	Married	479 (95.23)	87	216	176	38.663	<0.001
	Divorced/widowed/others	24 (4.77)	17	4	3		
Occupation	Farmer	47 (9.34)	21	17	9	32.879	<0.001
	Worker	48 (9.54)	9	24	15		
	State-owned enterprise/institution/civil servant	40 (7.95)	5	23	12		
	Employees in service industry	90 (17.89)	17	28	45		
	Retired personnel	110 (21.87)	25	47	38		
	Others(including freelancers and flexible employment workers)	168 (33.40)	27	81	60		
Family per-capita monthly income (RMB)	<2000	49 (9.74)	30	16	3	85.433	<0.001
	2000 ~ 4,999	100 (19.88)	40	54	46		
	5,000 ~ 9,999	201 (39.96)	29	100	72		
	>10,000	113 (22.47)	5	50	58		
Medical insurance	Uninsured	54 (10.74)	36	17	1	83.309	<0.001
	Insured	449 (89.26)	68	203	178		
Self-rated health status	Good/very good	217 (43.14)	30	89	98	25.358	<0.001
	Average	234 (46.52)	54	110	70		
	Bad/very bad	52 (10.34)	20	21	11		
CHAI		37.43 ± 5.80	33.92 ± 6.08	38.06 ± 4.60	38.68 ± 6.18	27.033	<0.001
Brief PETS		114.9 ± 11.51	128.5 ± 4.93	117.7 ± 7.93	103.6 ± 5.91	489.317	<0.001

**Table 4:** Comparison of scores of variables in three profiles.

Variables	Score	C1 (*n* = 104)	C2 (*n* = 220)	C3 (*n* = 179)	Multiple comparisons	*F*	*p*-value
CHAI	37.43 ± 5.80	33.92 ± 6.08	38.06 ± 4.60	38.68 ± 6.18	C1 < C2 < C3	27.033	<0.001
Knowledge	12.03 ± 2.57	10.60 ± 2.45	12.43 ± 2.38	12.36 ± 2.60	C1 < C2, C2 > C3	21.954	<0.001
Self-efficacy	14.42 ± 2.61	12.17 ± 1.63	14.55 ± 2.35	14.89 ± 2.71	C1 < C2 < C3	12.266	<0.001
Action	10.98 ± 1.93	9.32 ± 1.46	11.08 ± 1.67	11.44 ± 2.02	C1 < C2 < C3	21.059	<0.001
Brief PETS	114.94 ± 11.51	128.59 ± 4.93	117.71 ± 7.93	103.61 ± 5.91	C1 > C2 > C3	489.317	<0.001
Medical information	21.10 ± 2.98	23.06 ± 2.51	21.31 ± 2.99	19.69 ± 2.47	C1 > C2 > C3	51.522	<0.001
Medications	7.50 ± 1.45	9.22 ± 0.74	7.69 ± 1.21	6.25 ± 0.68	C1 > C2 > C3	324.454	<0.001
Medical appointments	6.79 ± 1.21	7.44 ± 1.00	7.35 ± 0.65	5.73 ± 1.12	C1 > C2 > C3	156.832	<0.001
Monitoring health	7.78 ± 1.40	9.35 ± 0.71	8.04 ± 1.12	6.53 ± 0.78	C1 > C2 > C3	314.848	<0.001
Role activity limitations	13.77 ± 2.20	15.18 ± 1.62	14.56 ± 1.69	11.98 ± 1.87	C1 > C2 > C3	150.587	<0.001
Physical and mental exhaustion	18.18 ± 1.98	20.31 ± 1.96	18.13 ± 1.45	17.02 ± 1.49	C1 > C2 > C3	143.008	<0.001
Diet	7.08 ± 1.48	8.58 ± 1.36	7.35 ± 1.06	5.87 ± 0.95	C1 > C2 > C3	215.044	<0.001
Exercise/physical therapy	7.40 ± 1.55	9.12 ± 0.75	7.68 ± 1.24	6.07 ± 1.00	C1 > C2 > C3	280.200	<0.001
Medical/healthcare expenses	15.24 ± 1.92	15.11 ± 1.78	14.98 ± 1.71	15.63 ± 2.17	C1 > C2, C3 > C2	6.013	<0.001
Difficulty with healthcare services	6.77 ± 1.27	7.30 ± 1.23	7.10 ± 1.17	6.06 ± 1.08	C1 > C2 > C3	54.066	<0.001
Medication side effects bother	3.35 ± 0.96	3.92 ± 0.87	3.53 ± 0.92	2.78 ± 0.74	C1 > C2 > C3	68.723	<0.001

### 3.6 Multivariate analysis of the latent profiles of TCM-HL in middle-aged and older adults patients with chronic diseases

Multiple logistic regression was conducted using the potential features of TCM-HL (C1 = 1, C2 = 2, C3 = 3) as dependent variables. The independent variables include all significant factors identified in the univariate analysis. The original scores were utilized for the the CHAI and Brief PETS. The coding for other variables is presented in Table 5. The results indicated (see Table 6) that, compared to C3, participants with lower per capita household income (<2000 or 2000–4,999) and a higher burden of disease treatment were more likely to be classified into C1 (OR = 104.263, 30.091, 1.864, *p*-value all<0.05). Patients lacking medical insurance were more likely to be classified into C1 and C2 (OR = 331.408, 180.779, both *p*-value<0.05). Married participants and those with a higher public health positive index were more likely to be classified into C3 (OR = 0.020, 0.874, both *p*-value<0.05). In comparison to C1, participants with lower education levels (primary school and below or junior high school), those engaged in the service industry, lower per capita household income (<2000 or 2000–4,999), and a higher disease treatment burden were more likely to be classified into C2 (OR = 0.084, 0.142, 0.136, 0.018, 0.041, 0.762, all *p*-value<0.05). Additionally, participants who were married and had a higher public health positive index were more likely to be classified into C2 (OR = 15.276, 1.106, both *p*-value<0.05).

**Table 5:** Assignment of independent variables in multiple logistic regression.

Variables	Assignment of variables
Age	45 ~ 55 = 1, 56 ~ 65 = 2, >65 = 3
Gender	Female = 1, Male = 2
Education level	Primary school and below = 1, junior high school = 2, high school and above = 3
Marital status	Married = 1, divorced/widowed/others = 2
Occupation	Farmer = 1, worker = 2, state-owned enterprise/institution/civil servant = 3, Employees in service industry = 4, retired personnel = 5, Others (including freelancers and flexible employment workers) = 6
Family per-capita monthly income (RMB)	<2000 = 1, 2000 ~ 4,999 = 2, 5,000 ~ 9,999 = 3, >10,000 = 4
Medical insurance	Uninsured = 1, insured = 2
Self-rated health status	Good/very good = 1, average = 2, bad/very bad = 3

**Table 6:** Multiple logistic regression analysis of latent categories.

Variable	*β*	SE	Wald *χ*^2^	*p*-value	OR(95% CI)
C1 vs. C3*					
Intercept	−69.440	7.470	88.420	<0.001	
Married (divorced/widowed/others)	−3.897	1.631	5.708	0.017	0.020 (0.001 ~ 0.497)
<2000 (>10,000)	4.647	1.365	11.588	<0.001	104.263 (7.181 ~ 1513.854)
2000 ~ 4,999 (>10,000)	3.064	1.086	9.819	0.002	30.091(3.578 ~ 253.037)
Medical insurance (yes)	5.803	2.262	6.584	0.010	331.408 (3.938 ~ 27891.397)
Brief PETS	0.595	0.053	127.079	<0.001	1.864 (1.669 ~ 2.081)
The consumer health activation index	0.623	0.056	122.407	0.017	0.874 (0.787 ~ 0.970)
C2 vs. C3*					
Intercept	−38.937	4.572	72.532	<0.001	
Medical insurance (Yes)	5.197	2.181	5.680	0.017	180.779 (2.517 ~ 12985.411)
Brief PETS	0.350	0.038	85.840	<0.001	1.420 (1.318 ~ 1.529)
C2 vs. C1*					
Intercept	30.503	5.901	26.721	<0.001	
Primary school and below (junior college and above)	−2.477	0.687	13.002	<0.001	0.084 (0.022 ~ 0.323)
Junior high school (junior college and above)	−1.954	0.556	12.326	<0.001	0.142 (0.048 ~ 0.422)
Married (divorced/widowed/others)	2.726	1.239	4.841	0.028	15.276 (1.347 ~ 173.269)
Employees in service industry [others(including freelancers and flexible employment workers)]	−1.997	0.745	7.189	0.007	0.136 (0.032 ~ 0.584)
<2000 (>10,000)	−4.019	1.056	14.484	<0.001	0.018(0.002 ~ 0.142)
2000 ~ 4,999 (>10,000)	−3.192	0.940	11.529	<0.001	0.041 (0.007 ~ 0.259)
The consumer health activation index	0.101	0.042	5.723	0.017	1.106 (1.018 ~ 1.201)
Brief PETS	−0.272	0.041	43.477	<0.001	0.762 (0.702 ~ 0.826)

*For the reference group; the highest assignment was used as the control for all independent variables.

## 4 Discussion

### 4.1 The overall TCM-HL level of middle-aged and older adults patients with chronic diseases

In this study, the TCM-HL score among middle-aged and older adults patients with chronic diseases was found to be (62.79 ± 14.42), with a compliance rate of 28.03%. These findings indicate an overall low level of health literacy, which is lower than previously reported results (23, 38). This discrepancy may be related to the characteristics of the study population. Unlike previous studies that primarily focused on healthy individuals (23) or patients with a single chronic disease (38), the majority of participants in this study were diagnosed with two or more chronic diseases(398/503, 79.13%). The results suggest an association between chronic diseases and residents’ health information literacy, with older adults individuals in poor health tending to have lower levels of health literacy (39). Patients with chronic or multiple diseases generally display lower health literacy levels compared to those with either a single chronic disease or no chronic disease (40, 41). Accordingly, greater attention should be directed towards improving the health literacy of older adults patients with two or more chronic diseases in the context of Traditional Chinese Medicine.

The basic concept of TCM, appropriate methods of public heath, TCM-based healthy lifestyle, health culture common sense of TCM and TCM information understanding ability of middle-aged and older adults patients with chronic diseases were 37.97%, 36.78%, 22.07%, 28.03% and 43.14%, respectively. This data indicates that patients possess a relatively strong ability to understand TCM information, while their TCM-based healthy lifestyle and health culture common sense of TCM are comparatively weaker. This suggests that middle-aged and older adults patients with chronic diseases are proficient in grasping the fundamental concepts and knowledge of TCM. However, there is a notable deficiency in their understanding of TCM cultural common sense, and a robust TCM-based healthy lifestyle has yet to be established. A pronounced gap exists between the acquisition of TCM knowledge and its practical application. Given the prolonged duration of chronic diseases in this demographic, which is often accompanied by recurrent episodes (42), effective TCM health management poses a considerable challenge. Accordingly, enhancing the overall TCM-HL among middle-aged and older adults patients with chronic diseases necessitates more than the mere transmission of knowledge; it requires active patient engagement. Diversifying the avenues for TCM health education and popularization, such as instructing patients in functional exercise, acupoint massage, and wellness exercises is essential. This approach aims to help patients sustain exercise and health habits, ultimately fostering a subtle integration of TCM health behaviors into their daily lives.

### 4.2 TCM-HL among middle-aged and older adults patients with chronic diseases: three latent profiles

Through LPA, this study reveals that the TCM-HL of middle-aged and older adults patients with chronic diseases is not homogeneous; rather, it exhibits a multidimensional and heterogeneous potential category structure. The findings categorize health literacy into three distinct groups: C1, “Low TCM-HL”; C2, “Moderate TCM-HL”; and C3, “High TCM-HL”.

C1 (n = 104, 20.68%) demonstrated a low overall level of TCM-HL, with an average score of 1.13 ± 1.33. The dimension scores were notably low: 1.08 ± 1.27 (basic concepts of TCM), 1.28 ± 1.58 (appropriate methods of public health), 1.05 ± 1.27 (TCM-based healthy lifestyle), 1.16 ± 1.23 (health culture common sense of TCM), and 1.13 ± 1.11 (understanding of TCM information). This group of patients encountered substantial obstacles in obtaining, understanding, evaluating, and applying information related to TCM health care and disease management. In contrast, C2 (*n* = 220, 43.74%) exhibited a moderate overall level of TCM-HL, with an average score of 1.70 ± 1.38. Patients in this group possess some understanding of basic TCM knowledge; however, they appeared to have deficiencies in converting this knowledge into effective behaviors and critically evaluating the authenticity of TCM information. C3 (*n* = 179, 35.59%) was comparatively high, with an average score of 2.03 ± 1.37. The scores across different dimensions were as follows: 2.28 ± 1.18 (basic concepts of TCM), 2.13 ± 1.61 (appropriate methods of public health), 1.83 ± 1.34 (TCM-Based healthy lifestyle), 1.62 ± 1.16 (health culture common sense of TCM), and 2.45 ± 1.25 (understanding of TCM information). The high literacy group not only mastered the core concepts of TCM but also adeptly applied this knowledge to guide their daily diet, emotional regulation, and exercise conditioning, effectively integrating it with modern medical management. This classification highlights the necessity of differentiated strategies in intervention practices. For the low-literacy group, it is essential to begin with basic knowledge and information acquisition channels, while for the moderate and high-literacy groups, the focus should be on skill enhancement and behavior consolidation.

### 4.3 Personal factors influencing TCM-HL in middle-aged and older adults patients with different latent characteristics of chronic diseases

The results of this study indicate that education level, marital status, per capita household income, medical insurance, and occupation are associated with the potential characteristics of TCM-HL among middle-aged and older adults patients with chronic diseases. Specifically, lower educational attainment, limited economic resources, lack of medical insurance, absence of a partner and employment in the service industry correlate with diminished TCM-HL. This finding aligns with previous research that identifies low education levels and low income as factors associated with inadequate TCM-HL among residents (23, 43). This phenomenon may be explained by the fact that educational attainment not only influences an individual’s ability to access, comprehend, and apply health information but also impacts the depth of understanding of TCM culture and concepts. The theoretical framework of TCM encompasses unique cultural connotations and cognitive patterns, necessitating specific cognitive abilities and cultural foundations for effective assimilation. Accordingly, individuals with lower educational levels may encounter greater challenges in understanding and applying TCM health knowledge, which may help explain the lower TCM-HL among patients.

Secondly, economic capacity and medical insurance jointly constitute the material security dimension associated with health literacy. Economic conditions may limit individuals’ ability to access paid health services, purchase relevant literature, or participate in health education activities. Meanwhile, the absence of health insurance may be related to reduced access to formal TCM services, which in turn may be associated with fewer opportunities to engage with and learn accurate TCM health knowledge. Studies indicate that (44) economic pressure is related to health literacy inequality, particularly in chronic disease management, where financial burdens correlate with patients’ health information-seeking behaviors and self-management capabilities. Individuals with high health literacy tend to demonstrate a deeper understanding of the healthcare system and are more likely to adhere to medical advice (45). Those without health insurance are often associated with economic hardship, prompting many to opt for short-term or passive medical care, which results in limited access to and experience with systematic and standardized TCM services, as well as a lack of opportunities and motivation to enhance TCM-HL. Improving TCM-HL among this vulnerable population necessitates expanding health insurance coverage, incorporating more standardized chronic disease management programs into reimbursement, and reducing economic barriers.

Furthermore, our research indicates that the level of TCM-HL among middle-aged and older adults patients with chronic diseases in the service industry is relatively low. The service industry is characterized by high work intensity, long hours, and unstable income, which are related to time poverty and significant economic constraints (46). In such circumstances, individuals are more likely tend to engage in passive learning and often do not actively pursue health management; only those with ample free time are more likely to engage in proactive health knowledge acquisition. Therefore, it is essential to consider their professional characteristics and provide flexible TCM services, such as Night-Time Outpatient, simplified conditioning programs (e.g., decoctions, granules), short-term health lectures in the workplace, or guidance on exercises during work breaks. The association of marital status may operate through social support mechanisms. Married individuals typically receive more informational support and behavioral encouragement from their spouses, which may facilitate more active participation in health management and enhance health literacy. Social support networks serve as a critical intermediary factor in promoting health literacy and health behaviors among middle-aged and older adults individuals (47). Accordingly, we should assist single middle-aged and older adults patients with chronic diseases in establishing social networks to improve their TCM-HL levels.

### 4.4 Differences in experience with treatment and self-management among middle-aged and older adults patients with chronic diseases based on different TCM-HL potential profile characteristics

The results of this study indicate a statistically significant difference in the disease treatment burden scores among middle-aged and older adults patients with chronic diseases, based on varying profiles of TCM-HL (*p* < 0.001). This suggests that different patient types exhibit distinct self-management behavior experiences. Specifically, the total disease treatment burden score for the C1was higher compared to the C2 and C3. This finding suggests a negative association between TCM-HL levels and disease burden; middle-aged and older adults patients with chronic diseases who possess lower TCM-HL demonstrate poorer disease management experiences. This observation aligns with the conclusions drawn from general health literacy research, while also incorporating the unique contextual factors associated with TCM (48).

Higher levels of TCM-HL are associated with better understanding of the pathogenesis of chronic diseases and rehabilitation expectations that more closely align with disease patterns. This, in turn, mitigates the cognitive burden associated with misunderstandings or inappropriate expectations. TCM posits that “keeping healthy Qi and preventing evil pathogens”. Patients with high-quality health literacy are more likely to proactively engage in dietary therapy, guidance, emotional regulation, and other methods to support healthy qi, alleviate symptoms, and show corresponding reductions in physical and psychological symptoms burden. Conversely, patients with low literacy may be more prone to blindly following trends, misusing health supplements, or demonstrating poor adherence to standard treatments due to a lack of information or misunderstandings, which can exacerbate their health burden. The health concepts of ‘man-nature harmony’ and ‘body-spirit syncretism’ in TCM, along with practical techniques such as self-acupoint massage, dietary therapy, and Baduanjin, may provide patients with effective self-efficacy tools. Patients with high TCM-HL are more adept at applying these theories and techniques to integrate disease management into their daily lives, which may contribute to an active, internalized, and culturally recognized management model and correspond to improved management experiences and quality of life. In contrast, patients with lower literacy tend to be overly reliant on medical institutions and personnel, with lower self-efficacy, and may report feelings of helplessness and frustration when faced with fluctuations in their condition, patterns assiciated with unsatisfactory management experiences (49). Improving health literacy facilitates patients to effectively understand and apply health information, boosts their initiative in disease management, and increases the likelihood of adhering to medical advice (50). Accordingly, providing diverse and personalized self-management tools in TCM, such as health regimen based on Twenty-Four Solar Terms and emotional regulation, may offer patient’s alternatives beyond conventional medical advice, enrich their repertoire of self-management strategies, and strengthen their ability to cope with the challenges of daily disease management.

### 4.5 Middle-aged and older adults patients with chronic diseases who have high TCM-HL levels exhibit better consumer health activation index

The results of this study indicate that TCM-HL is positively correlated with self-management ability and the public health positive index. Middle-aged and older adults patients with high levels of TCM-HL tend to have a higher consumer health activation index, underscoring the value of TCM empowerment. This finding aligns with the results of Haering et al., which demonstrated that patients with higher health literacy exhibited elevated consumer health activation index (29). TCM-HL not only pertains to disease management but also serves as a resource for promoting health autonomy. Patients with elevated TCM-HL are more likely to integrate Chinese medicine self-cultivation knowledge into their daily lives—such as through dietary restraint, regular living activity, and moderate exercise—which may cultivate a positive self-management behavior model. This successful self-management experience, combined with the appreciation of philosophical concepts like the ‘man-nature harmony’ in TCM, may further foster a positive attitude towards life and health. This, in turn, manifests as a higher consumer health activation index, characterized by a stronger sense of health responsibility, more proactive health information seeking, and more affirmative health values (51). Accordingly, to enhance the capacity for health information acquisition among middle-aged and older adults patients with chronic diseases, a peer support model may be adopted. Patients possessing high levels of TCM-HL can be invited to provide face-to-face practical knowledge and emotional support, thereby helping to build their confidence, improve their health literacy, and stimulate their enthusiasm for participation in disease management.

## 5 Limitations

This study has several limitations that may affect both internal and external validity. Firstly, the sample is exclusively composed of middle-aged and older adults patients from tertiary Chinese medicine hospital and its nine affiliated community health service centers in Shenzhen, Guangdong Province. Although primary medical institutions are considered, patients from rural and urban areas, as well as those receiving treatment at secondary medical institutions or in economically underdeveloped areas, are not included. This omission may limit the generalization of the research findings. Furthermore, since the samples originate from a TCM hospital and its affiliated community health service centers in Shenzhen—a national comprehensive reform pilot area for TCM—there may be enhanced infrastructure and health knowledge related to TCM, which could positively influence patients’ health literacy regarding TCM. Secondly, this study employs a cross-sectional survey design, capturing data at a single point in time. Future longitudinal studies are necessary to assess the trajectory of participants’ health literacy, thereby verifying potential causal relationships among TCM-HL, treatment experiences, treatment and Self-management, consumer health activation index, and related influencing factors. Thirdly, all data were collected through patients’ self-perceived, self-administered questionnaires, which may be influenced by common methodological biases. Finally, although other tools, such as the TCM-HL utilized in this study, have been validated in the Chinese population and demonstrate good reliability and validity, their use in international research remains limited. This constraint may hinder the comparability of research findings across diverse cultural and healthcare contexts. Future research should prioritize the incorporation of tools that are widely recognized and utilized internationally to enhance cross-cultural applicability and validity.

## 6 Conclusion

In summary, this study demonstrates significant categorical differences in TCM-HL among middle-aged and older adults patients with chronic diseases. High TCM-HL is identified as a crucial protective factor linked to a lower disease burden, enhanced self-management, and more positive health perceptions. Enhancing TCM-HL within this demographic, particularly through tailored educational interventions for distinct characteristic categories, may serve as an effective strategy to optimize chronic disease management and alleviate the societal burden of these conditions.

## Data Availability

The raw data supporting the conclusions of this article will be made available by the authors, without undue reservation.
